# Active Cognitive Lifestyle Is Associated with Positive Cognitive Health Transitions and Compression of Morbidity from Age Sixty-Five

**DOI:** 10.1371/journal.pone.0050940

**Published:** 2012-12-12

**Authors:** Riccardo E. Marioni, Michael J. Valenzuela, Ardo van den Hout, Carol Brayne, Fiona E. Matthews, MRC Cognitive Function and Ageing Study

**Affiliations:** 1 Department of Public Health and Primary Care, University of Cambridge, Cambridge, United Kingdom; 2 Regenerative Neuroscience Group, Brain and Mind Research Institute, University of Sydney, Sydney, New South Wales, Australia; 3 Department of Statistical Science, University College London, London, United Kingdom; 4 Medical Research Council Biostatistics Unit, Cambridge, United Kingdom; The University of Hong Kong, Hong Kong

## Abstract

**Background:**

Three factors commonly used as measures of cognitive lifestyle are education, occupation, and social engagement. This study determined the relative importance of each variable to long term cognitive health in those with and without severe cognitive impairment.

**Methods:**

Data came from 12,470 participants from a multi-centre population-based cohort (Medical Research Council Cognitive Function and Ageing Study). Respondents were aged 65 years and over and were followed-up over 16 years. Cognitive states of no impairment, slight impairment, and moderate/severe impairment were defined, based on scores from the Mini-Mental State Examination. Multi-state modelling was used to investigate links between component cognitive lifestyle variables, cognitive state transitions over time, and death.

**Results:**

Higher educational attainment and a more complex mid-life occupation were associated with a lower risk of moving from a non-impaired to a slightly impaired state (hazard ratios 0.5 and 0.8), but with increased mortality from a severely impaired state (1.3 and 1.1). More socially engaged individuals had a decreased risk of moving from a slightly impaired state to a moderately/severely impaired state (0.7). All three cognitive lifestyle variables were linked to an increased chance of cognitive recovery back to the non-impaired state.

**Conclusions:**

In those without severe cognitive impairment, different aspects of cognitive lifestyle predict positive cognitive transitions over time, and in those with severe cognitive impairment, a reduced life-expectancy. An active cognitive lifestyle is therefore linked to compression of cognitive morbidity in late life.

## Introduction

The ageing of modern nations is set to radically alter the nature of society, arguably with no greater impact than on the prevalence and cost implications of dementia. Currently, 800,000–900,000 persons suffer from dementia in the UK, with an annual estimated cost to the economy of £22–23 billion [1]. Whilst mortality rates for stroke, heart disease and other common causes of death declined between 2000 and 2008, the equivalent figure for Alzheimer's dementia rose by 66% [2]. By 2050, dementia prevalence is expected to nearly quadruple to over 115 million worldwide [3] and at the same time the proportion of tax-paying workers to retirees will fall by 40% [4]. Projections in Australia suggest that assuming the *status quo*, dementia-related care in 2060 would consume the entire current health budget [5], clearly an unsustainable scenario that will force unenviable health decisions by policy makers.

Given these forecasts, identification of modifiable factors that reduce the risk of developing severe cognitive impairment or encourage cognitive recovery from *mild cognitive impairment* (MCI) to a non-cognitively impaired state are highly sought after. MCI describes a stage of intermediate cognitive dysfunction where the risk of ‘conversion’ to dementia is increased but is by no means guaranteed [6]. Indeed, it is possible for individuals to revert from a diagnosed MCI state back to a normal, non-impaired cognitive state over time [6].

There is considerable evidence to support a link between an active cognitive lifestyle and a decreased risk of cognitive decline and dementia [7], [8]. Previously, we have found that greater cognitive lifestyle, denoted by education, occupational complexity and late-life social engagement, was predictive of decreased dementia incidence over and above other known risk factors [9]. However, this paper did not explore the link between cognitive lifestyle and cognitive decline. Furthermore, whilst each individual factor was itself not independently predictive of lower risk, the combination of a higher level of education plus either a more complex occupation or late life social engagement decreased prospective dementia risk by 40%. This may explain why reviews of these components in isolation have been mixed [10]–[12]. Active participation in any single cognitive lifestyle factor appears not to be sufficiently powerful to modify dementia risk, but ongoing engagement throughout the lifespan may accumulate and provide additive or synergistic value.

Whether the same is true in models of cognitive decline is not clear. Recently, we showed that an active cognitive lifestyle was associated with a decreased risk of cognitive decline, an increased chance of cognitive recovery, but an increased mortality risk from the most severely impaired cognitive state [13]. However, this paper did not break down cognitive lifestyle into its individual components but instead used an overall summary score, measured as a weighted linear combination of education, occupation and social engagement.

We now extend this work by investigating the independent and combined impact of cognitive lifestyle factors on transitions between the following clinically relevant cognitive states: non-impaired cognitive health, mild impairment and moderate-to-severe impairment, and thereafter, death. Such distinctions are important because in contrast to the protective findings in those with intact cognition, high levels of engagement in education or occupation have been suggested to decrease lifespan and accelerate clinical decline after the onset of dementia or severe impairment [14], [15]. Furthermore, no study has yet to establish whether specific aspects of an active cognitive lifestyle alter the chance of reverting from the intermediate MCI state back to cognitive health.

In this analysis we used multi-state modelling to assess the relationship between separate and combined measures of cognitive lifestyle to forward and backward transitions between cognitive states over time in a population-based cohort of the elderly.

## Methods

### Ethics Statement

MRC CFAS has multi-centre research ethics committee's approval and ethical approval from the relevant local research ethics committees.

### Study Population

The Medical Research Council Cognitive Function and Ageing Study (MRC CFAS) is a multi-centre study on over 18,000 persons from across four urban and two rural centres in England and Wales including institutions [16]. A standardised study design was used in five of the centres. This incorporated a two-phase sampling process with a screening interview followed by an assessment interview. Participants were identified from Family Health Service Authority lists and were 65 years and over at the index date for each centre. Initial meetings with participants took place between 1989 and 1993.

This analysis used data from the five centres with a standardised design: Cambridgeshire (n = 2,601), Gwynedd (n = 2,625), Newcastle (n = 2,524), Nottingham (n = 2,514), and Oxford (n = 2,740). The total sample size was 13,004. Persons were excluded from the analysis if they only had a single data point i.e. no cognitive transitions were recorded (n = 159), if they did not have a baseline cognitive measurement (n = 246), or if they had any missing covariate data (mid-life occupational status, n = 320; late-life social engagement, n = 83). This left an analysis sample of 12,470. Up to ten waves of cognitive data, collected over a 16-year follow-up period were available for the analysis and 9,285 deaths were observed in the sample.

### Cognitive assessment

Cognitive ability was assessed at each wave using the Mini-Mental State Examination (MMSE) [17], which is a widely used, brief screening instrument for dementia with an integer scoring range of 0 to 30. Cognitive states were assigned as follows: no impairment (MMSE 27–30); mild impairment (23–26); moderate-to-severe impairment (0–22). These groupings centre around the mild impairment category, which is based on a figure from Stephan et al. [18]. They showed that within a population-based setting the MMSE is as effective a predictor of dementia risk as more complex measures of MCI. In a previous analysis [13] that assessed cognitive lifestyle as a single measure, the most impaired group was split into moderate (18–22) and severe impairment (0–17). As no significant associations were observed between these states and for computational efficiency these groups were merged.

### Cognitive lifestyle variables

Three cognitive lifestyle variables were assessed: education, mid-life occupation, and late-life social engagement. In a previous MRC CFAS analysis, a combination of these measures was found to correlate highly with the total score on the Lifetime of Experiences Questionnaire [9]. Education level in young adulthood was split into three groups: 0–9 years; 10–11 years; >11 years. Mid-life occupational complexity was recorded as the main occupation in terms of years worked. This was then recoded using two systems: social class grouping (from I to VI) and socio-economic grouping (from 11 to 150) [9]. Social engagement in later life was assessed by asking three questions about the subject's contact with relatives and neighbours, and the frequency that they attended meetings, such as social groups and evening classes. These questions were assessed using a 3-point Likert scale. Sex-specific tertiles for occupation and social engagement were generated to investigate high, medium and low groupings.

### Statistical Analysis

The associations between the predictor variables and cognitive decline were investigated by using multi-state models [19] with three living states (no impairment; mild impairment; moderate-to-severe impairment) and death as an absorbing state. Forward transitions, which represent movement to a more impaired state, were allowed between adjacent cognitive states and from all cognitive states to death. A back transition (cognitive recovery) was allowed from the mild impairment state to the non-impaired state. Due to a lack of observed transitions and our previously observed null associations in a five state model [13], recovery from moderate-to-severe impairment was not allowed. Where such transitions were observed, they were treated as measurement error (misclassification). Right censoring was used to account for sample attrition.

There are several benefits in using a multi-state model to analyse longitudinal cognitive data. These include: the ability to model death, decline and recovery within the same model; allowing covariate effects to vary for each transition, and modelling measurement error via state misclassification. State misclassification is modelled via a hidden Markov model, which considers an individual's cognitive state at each wave as a potentially misclassified measure of their true level.

Three initial models were run with sex and year of birth minus 1900 (birth cohort effect) as fixed covariates, age as a time dependent covariate, and one of education, mid-life occupation and late-life social engagement as the fixed covariate of interest. Occupation and social engagement were then added sequentially to the model that contained education with likelihood ratio tests indicating the full model with all three cognitive lifestyle predictors to be the best fit.

From this model, life expectancies were calculated separately for men and women in the youngest birth cohort (age 65 at baseline i.e., born in 1927). Total residual life expectancy is defined as the sum of occupancy times in each living state. Data were analysed using R version 2.10.1 [19] and the ‘msm’ package in R [20]. For full details of the multi-state model and life expectancy calculations see Appendix S1.

## Results

Table 1 shows the baseline characteristics of the study population split by education level. The distributions of age and sex were similar across groups with the mean age being around 75 years and with 60% of the population being women. Those with the lowest educational attainment (<10 years) tended to have a less complex occupation in mid-life compared to those with the highest educational attainment (>11 years). Nineteen percent of those in the least educated group were in the most complex mid-life occupation group compared to 61% of those in the highly educated group. By contrast, there was very little difference in late-life social engagement by educational attainment. A higher proportion of people with a low level of education had a MMSE score indicating moderate-to-severe impairment (0–22) compared to those with a high level of education (20% versus 6%).

**Table 1:** Summary of population characteristics at baseline.

	Education					
	0–9 years		10–11 years		>11 years	
	(n = 7,884)		(n = 2,673)		(n = 1,913)	
Age (s.d.)	75.3	(7.0)	74.7	(6.6)	75.2	(6.7)
Sex – n (%)						
Male	3236	(41.0)	1023	(38.1)	767	(40.0)
Female	4648	(59.0)	1650	(61.9)	1146	(60.0)
Mid-life occupation complexity – n (%)						
Tertile 1 (Low)	3571	(45.3)	898	(33.6)	409	(21.4)
Tertile 2 (Medium)	2814	(35.7)	804	(30.1)	332	(17.4)
Tertile 3 (High)	1499	(19.0)	971	(36.3)	1172	(61.3)
Late-life social engagement – n (%)						
Tertile 1 (Low)	2926	(37.1)	949	(35.6)	682	(35.7)
Tertile 2 (Medium)	2383	(30.2)	780	(29.2)	552	(28.9)
Tertile 3 (High)	2575	(32.7)	944	(35.3)	679	(35.5)
MMSE group – n (%)						
No impairment (27–30)	3616	(45.9)	1599	(59.8)	1383	(72.3)
Mild impairment (23–26)	2688	(34.1)	785	(29.4)	411	(21.5)
Moderate-to-severe impairment (0–22)	1580	(20.0)	289	(10.8)	119	(6.2)

**Figure 1:**
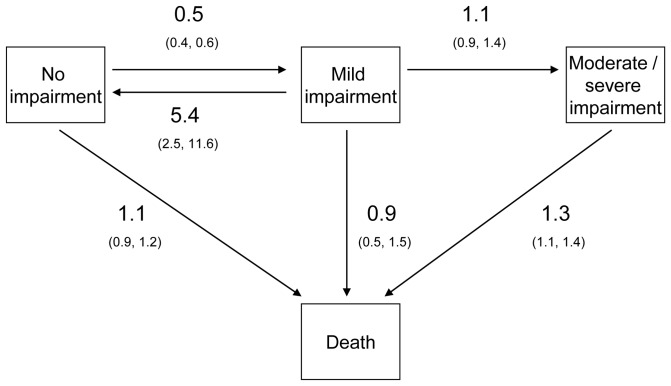
Hazard ratios (95% confidence intervals) for high versus low education on the cognitive and mortality transitions. Results adjusted for age, sex, occupational complexity, and level of social engagement. Cognitive states defined as: no impairment (MMSE 27–30), mild impairment (23–26), and moderate-to-severe impairment (0–22).

Output from the best fitting multi-state model with all three cognitive lifestyle variables is presented in Table 2; the results for education after covariate adjustment for age, sex, occupation and social engagement are presented in Figure 1. A high level of education and a complex mid-life occupation were associated with a decreased risk of cognitive decline to mild impairment (hazard ratios and 95% confidence intervals for the high versus low groups: 0.5 [0.4, 0.6] and 0.8 [0.6, 1.0], respectively). Both were also associated with an increased mortality risk from the moderate-to-severely impaired state (HRs 1.3 [1.1, 1.4] and 1.1 [1.0, 1.3]). A high level of late-life social engagement was linked to a decreased risk of decline from mild to moderate-to-severe impairment (HR 0.7 [0.6, 0.8]) and with a decreased mortality risk for those with no impairment (HR 0.9 [0.8, 1.0]). All three cognitive lifestyle variables were linked to cognitive recovery - the positive transition from mild impairment back to no impairment (HR education 5.4 [2.5, 11.6], occupation - medium versus low complexity 2.2 [1.1, 4.5], social engagement 0.7 [0.6, 0.8]). Compared to the models where each cognitive lifestyle term was entered separately, there were little differences in the magnitudes or the statistical significance of the associations (Table S1).

**Table 2:** Hazard ratios and 95% confidence intervals for the co-adjusted effects of cognitive lifestyle on cognitive decline and mortality.

	Cognitive lifestyle covariate					
	Education	Education	Occupational complexity	Occupational complexity	Social engagement	Social engagement
Transition	(Medium vs. Low)	(High vs. Low)	(Medium vs. Low)	(High vs. Low)	(Medium vs. Low)	(High vs. Low)
State 1 - State 2	**0.6 (0.5, 0.8)**	**0.5 (0.4, 0.6)**	0.9 (0.7, 1.1)	**0.8 (0.6, 1.0)**	1.0 (0.8, 1.3)	0.9 (0.8, 1.2)
State 1 - Death	1.0 (0.9, 1.2)	1.1 (0.9, 1.2)	1.0 (0.8, 1.2)	0.9 (0.8, 1.1)	1.0 (0.9, 1.2)	**0.9 (0.8, 1.0)**
State 2 - State 1	**2.6 (1.5, 4.8)**	**5.4 (2.5, 11.6)**	**2.2 (1.1, 4.5)**	1.8 (0.9, 3.7)	0.9 (0.4, 1.8)	**2.3 (1.2, 4.5)**
State 2 - State 3	0.9 (0.8, 1.1)	1.1 (0.9, 1.4)	1.0 (0.8, 1.1)	0.9 (0.8, 1.1)	**0.8 (0.7, 1.0)**	**0.7 (0.6, 0.8)**
State 2 - Death	1.0 (0.7, 1.4)	0.9 (0.5, 1.5)	1.1 (0.8, 1.4)	0.8 (0.5, 1.1)	1.0 (0.8, 1.3)	0.9 (0.7, 1.2)
State 3 - Death	1.1 (1.0, 1.2)	**1.3 (1.1, 1.4)**	1.0 (0.9, 1.1)	**1.1 (1.0, 1.3)**	1.0 (0.9, 1.1)	1.0 (0.9, 1.1)

State 1 (no impairment): MMSE 27–30, State 2 (mild impairment): MMSE 23–26, State 3 (moderate-to-severe impairment): MMSE 0–22.

The life expectancies for men and women are presented in Table 3. A 65 year old man (born in 1927) in the lowest education, occupation, and social engagement groups has an estimated total residual life expectancy of 12.8 years, with 8.5 years of that time spent without cognitive impairment. By contrast, a 65 year old man in the highest education, occupation, and social engagement groups has a total residual life expectancy of 15.9 years (14.3 years without cognitive impairment). This represents a 24% increase in both total residual life expectancy and the proportion of life expectancy without cognitive impairment for those with an active cognitive lifestyle. The corresponding life expectancies for women showed a 22% increase in total residual life expectancy and a 26% increase in life expectancy without cognitive impairment for those with an active cognitive lifestyle.

**Table 3:** Total residual and non-cognitively impaired life expectancies for individuals aged 65, 75, and 85 years in 1992.

	Total residual life expectancy in years (95% CI)											
	Low education, non-complex occupation, low social engagement				Medium education, medium-complex occupation, medium social engagement				High education, complex occupation, high social engagement			
Age in 1992	men		women		men		women		men		women	
65	12.8	(10.4, 15.6)	17.2	(13.6, 21.1)	13.5	(10.7, 16.9)	18.4	(14.1, 23.4)	15.9	(13.1, 19.0)	21.0	(16.6, 25.9)
75	7.6	(5.9, 9.7)	10.6	(7.7, 13.8)	7.8	(6.0, 9.9)	11.0	(8.3, 14.1)	8.8	(6.9, 11.0)	11.8	(9.2, 14.9)
85	3.3	(2.3, 4.5)	4.6	(3.1, 6.4)	3.3	(2.3, 4.5)	4.7	(3.3, 6.5)	3.5	(2.5, 4.7)	4.8	(3.4, 6.4)

	Non-cognitively impaired life expectancy in years (95% CI)											
	Low education, non-complex occupation, low social engagement				Medium education, medium-complex occupation, medium social engagement				High education, complex occupation, high social engagement			
Age in 1992	men		women		men		women		men		women	
65	8.5	(7.2, 10.0)	10.1	(8.3, 11.9)	10.7	(9.0, 12.8)	13.2	(10.8, 15.9)	14.3	(12.3, 16.2)	18.0	(15.0, 20.6)
75	4.2	(3.4, 5.0)	5.4	(4.0, 6.6)	4.7	(3.9, 5.6)	6.3	(5.0, 7.5)	5.5	(4.6, 6.6)	7.4	(6.1, 8.8)
85	1.3	(0.9, 1.6)	1.7	(1.1, 2.2)	1.4	(1.0, 1.7)	1.9	(1.4, 2.4)	1.6	(1.2, 2.0)	2.2	(1.7, 2.7)

## Discussion

Using data from 16 years of follow-up of 12,470 persons aged over 65 years and drawn from five centres across England and Wales, we found evidence linking an active cognitive lifestyle with compression of cognitive morbidity. Increased education and a more complex mid-life occupation were associated with a decreased risk of decline from no impairment to mild impairment and a reduced time spent with moderate-to-severe impairment before death. Late-life social engagement was associated with a reduced risk of decline from mild to moderate-to-severe impairment and with a lower mortality risk for those with no impairment. All three cognitive lifestyle variables increased the chance of improvement from mild impairment back to no impairment. Life expectancy calculations complement these findings by showing an extended total residual and cognitive-impairment-free lifetime for those with an active cognitive lifestyle.

While two of the cognitive lifestyle factors are potentially modifiable (education and social engagement), the mechanisms through which they exert a positive effect on cognitive transitions in late-life are not known. Our results suggest that little additional information is gained by adding mid-life occupation to a cognitive lifestyle model that includes education. Significant results were observed on transitions from no impairment to mild impairment and vice versa, and from moderate-to-severe impairment to death for both education and mid-life occupation but with greater effect sizes for the former variable. This suggests an important role for education in building a platform for success in later life, over and above the cognitive benefits that result from an intellectually demanding occupation and any subsequent impact on social structure and economic resources. Furthermore, if mid-life occupation did have a significant influence on social structure then one might expect to see similar covariate effects on the state transitions for social engagement as occupational status, but different effects were observed. Given this, our data suggest different pathways of cognitive protection for different aspects of the cognitive lifestyle construct.

Neurocompensation as opposed to neuroprotection has been proposed as a plausible biological mechanism linking education to protection from cognitive decline and dementia. One study that included a subset of 414 individuals from MRC CFAS showed that individuals with a low level of education and moderate-to-severe Alzheimer pathology were more likely to be demented just prior to death, compared to those with high levels of education and the same degree of pathology [21]. Also based on the MRC CFAS brain series, we have shown that a more active cognitive lifestyle is associated with a regional increase in cortical thickness in Brodmann area 9 and 10 of the frontal cortex, accompanied by greater neuronal density, suggesting this brain region may have an important role in mediating neurocompensation [22]. Failure of neuronal compensation may best describe our finding of a heightened mortality risk in those with high education when exhibiting moderate-to-severe cognitive impairment. Education may delay the onset of cognitive decline via compensatory mechanisms, but once neuropathological burden becomes too great and overwhelms the individual's compensatory mechanisms, a more rapid transition to death occurs.

Our findings were based on the combination of a unique epidemiological resource and a multi-state modelling approach to data analysis. CFAS is a large multi-centre population-based cohort with regular participant follow-up over 16 years. However, as in any longitudinal study of cognitive ageing, there is subject attrition due to death and dropout. A multi-state model is well suited to address these issues [19], [23]. Furthermore, state misclassification helps to account for indeterminacy when categorising an individual into a given clinical state, a common problem when distinguishing MCI or intermediate impairment from cognitive normalcy at one extreme, and dementia at the other. The multi-state approach also benefits from an explicit model of cognitive recovery, which here was found to be specific to certain cognitive lifestyle profiles.

Ideally, to further investigate these concepts life-course data would track cognitive change and participation in cognitive lifestyle variables from birth through to death. Consideration also needs to be given to genetic differences that may influence cognitive function. Recently, it has been shown that there is a detectable genetic contribution to cognitive change from childhood through to late-life however this is relatively small compared to the influence of environmental factors [24]. Another possible limitation was our use of the MMSE as a measure of cognition instead of domain-specific neuropsychological tests. Although it is mainly utilised as a dementia screening tool, the MMSE is frequently used to categorise subjects into risk groups in population-based cohorts and provides a readily interpretable and clinically relevant score. Here, the MMSE cut-points were based on published CFAS data [18], although it is recognised that categorising those with MMSE 23–26 as ‘mild impairment’ may be less accurate for individuals with lower natural cognitive ability. Our use of multi-state modelling was aimed at minimising any confound due to such misclassification.

In conclusion, our analytical approach and population-based data provides convincing evidence that an active cognitive lifestyle is associated with compression of cognitive morbidity in older persons. This could have significant implications for both public health and economic policy. For example, economic analyses have revealed a link between policies that promote a later age of retirement with population estimates of better memory function in late life [25]. In the context of the ageing of modern society, testing whether interventions based on stimulating cognitive lifestyle can translate into enhanced cognitive health is a challenge of international significance.

## Supporting Information

Appendix S1 Details of the multi-state model and life expectancy calculations.
(DOC)

Supporting Information S1 Diagram of the multi-state model. Supporting Information S1 Legend. There are six transition specific hazards, q_rs_(t), where r and s are contained within the cognitive state set (1, 2, 3, 4) and t represents time. A vector, **z**(t), containing the three cognitive lifestyle covariates plus age and sex is linked to each hazard via log-linear regression i.e. log [q_rs_(t)] = **β**_rs_^T^ **z**(t).
(TIF)

Table S1 Hazard ratios and 95% confidence intervals for the individual effects of education, mid-life occupation and late-life social engagement on cognitive decline and mortality. Table S1 Legend. State 1 (no impairment): MMSE 27–30, State 2 (mild impairment): MMSE 23–26, State 3 (moderate-to-severe impairment): MMSE 0–22.
(DOC)

## References

[1] Health Economics Research Centre (University of Oxford for the Alzheimer's Research Trust) (2010) Dementia 2010. Available: http://www.dementia2010.org/reports/Dementia2010Full.pdf. Accessed 2012 May 29.

[2] Thies W, Bleiler L (2011) Alzheimer's disease facts and figures. Alzheimers Dement 7: 208–44. 21414557 10.1016/j.jalz.2011.02.004

[3] Prince MJ, Jackson J, editors (2009) World Alzheimer Report 2009. London. Available: http://www.alz.co.uk/research/world-report. Accessed 2012 May 29.

[4] Circulated by The Hon.Wayne Swan MP Treasurer of the Commonwealth of Australia (2010) Intergenerational Report 2010: Australia to 2050: future challenges. Available: http://archive.treasury.gov.au/igr/igr2010/report/pdf/IGR_2010.pdf. Accessed 2012 May 29.

[5] Access Economics Pty Limited for Alzheimer's Australia (2009) Keeping Dementia Front of Mind: Incidence and prevalence 2009–2050. Available: http://www.fightdementia.org.au/common/files/NAT/20090800_Nat__AE_FullKeepDemFrontMind.pdf. Accessed 2012 May 29.

[6] Gauthier S, Reisberg B, Zaudig M, Petersen RC, Ritchie K, et al (2006) Mild cognitive impairment. Lancet 367: 1262–70. 16631882 10.1016/S0140-6736(06)68542-5

[7] Valenzuela MJ, Sachdev P (2006) Brain reserve and dementia: a systematic review. Psychol Med 36: 441–54. 16207391 10.1017/S0033291705006264

[8] Valenzuela MJ, Sachdev P (2006) Brain reserve and cognitive decline: a non-parametric systematic review. Psychol Med 36: 1065–73. 16650343 10.1017/S0033291706007744

[9] Valenzuela M, Brayne C, Sachdev P, Wilcock G, Matthews F (2011) Cognitive lifestyle and long-term risk of dementia and survival after diagnosis in a multicenter population-based cohort. Am J Epidemiol 173: 1004–12. 21378129 10.1093/aje/kwq476

[10] Fratiglioni L, Paillard-Borg S, Winblad B (2004) An active and socially integrated lifestyle in late life might protect against dementia. Lancet Neurol 3: 343–53. 15157849 10.1016/S1474-4422(04)00767-7

[11] Scarmeas N, Stern Y (2004) Cognitive reserve: implications for diagnosis and prevention of Alzheimer's disease. Curr Neurol Neurosci Rep 4: 374–80. 15324603 10.1007/s11910-004-0084-7 PMC3026565

[12] Sharp ES, Gatz M (2011) Relationship between education and dementia: an updated systematic review. Alzheimer Dis Assoc Disord 25: 289–304. 21750453 10.1097/WAD.0b013e318211c83c PMC3193875

[13] Marioni RE, van den Hout A, Valenzuela MJ, Brayne C, Matthews FE (2012) Active cognitive lifestyle associates with cognitive recovery and a reduced risk of cognitive decline. J Alzheimers Dis 28: 223–30. 21971400 10.3233/JAD-2011-110377

[14] Reuser M, Willekens FJ, Bonneux L (2011) Higher education delays and shortens cognitive impairment: a multistate life table analysis of the US Health and Retirement Study. Eur J Epidemiol 26: 395–403. 21337033 10.1007/s10654-011-9553-x PMC3109265

[15] Stern Y, Tang MX, Denaro J, Mayeux R (1995) Increased risk of mortality in Alzheimer's disease patients with more advanced educational and occupational attainment. Ann Neurol 37: 590–5. 7755353 10.1002/ana.410370508

[16] MRC CFAS (1998) Cognitive function and dementia in six areas of England and Wales: the distribution of MMSE and prevalence of GMS organicity level in the MRC CFA Study. Psychol Med 28: 319–35. 9572090 10.1017/s0033291797006272

[17] Folstein MF, Folstein SE, McHugh PR (1975) “Mini-mental state”. A practical method for grading the cognitive state of patients for the clinician. J Psychiatr Res 12: 189–98. 1202204 10.1016/0022-3956(75)90026-6

[18] Stephan BCM, Savva GM, Brayne C, Bond J, McKeith IG, et al (2010) Optimizing Mild Cognitive Impairment for Discriminating Dementia Risk in the General Older Population. Am J Geriatr Psychiatry 18: 662–73. 21491627 10.1097/jgp.0b013e3181e0450d

[19] R Development Core Team (2010) R: A Language and Environment for Statistical Computing. R Foundation for Statistical Computing.Vienna, Austria. Available: http://www.R-project.org. Accessed 2012 May 29.

[20] Jackson CH (2011) Multi-State Models for Panel Data: The msm Package for R. Journal of Statistical Software 38: 1–29.

[21] EClipSE (2010) Education, the brain and dementia: neuroprotection or compensation? Brain 133: 2210–6. 20826429 10.1093/brain/awq185

[22] Valenzuela MJ, Matthews FE, Brayne C, Ince P, Halliday G, et al (2011) Multiple Biological Pathways Link Cognitive Lifestyle to Protection from Dementia. Biol Psychiatry 71: 783–91. 22055015 10.1016/j.biopsych.2011.07.036

[23] van den Hout A, Matthews FE (2010) Estimating stroke-free and total life expectancy in the presence of non-ignorable missing values. J R Stat Soc Ser A Stat Soc 173: 331–49. 10.1111/j.1467-985X.2009.00610.x PMC2859253 20454440

[24] Deary IJ, Yang J, Davies G, Harris SE, Tenesa A, et al (2012) Genetic contributions to stability and change in intelligence from childhood to old age. Nature 482: 212–5. 22258510 10.1038/nature10781

[25] Rohwedder S, Willis RJ (2010) Mental Retirement. J Econ Perspect 24: 119–38. 20975927 10.1257/jep.24.1.119 PMC2958696

