# Supplementary material for: Active Cognitive Lifestyle Is Associated with Positive Cognitive Health Transitions and Compression of Morbidity from Age Sixty-Five
Source: PLoS One. 2012 Dec 12;7(12):e50940. doi: 10.1371/journal.pone.0050940 (PMC3521012; doi:10.1371/journal.pone.0050940)
Supplement: Appendix S1 — Details of the multi-state model and life expectancy calculations. (DOC) [file pone.0050940.s001.doc]

Appendix S1: Details of the multi-state model and life expectancy calculations.

The associations between the predictor variables and cognitive decline were investigated by using multi-state models. Four state models were used with three living states (no impairment; mild impairment; and moderate-to-severe impairment) and death as an absorbing state (Supporting Information S1). The probability of individual *i* being in state *s* at wave *w* is conditional on the state occupied and observed covariates, **z**(.), at the previous wave *s*_i_(*w-1*) and ***z***_i_(*w-1*) but not at any waves prior to this point. However, the model is not explicitly Markovian as the transition intensities are related to age, which is a time-dependent covariate. Neither is it a semi-Markov model as the time since entry into the state is not accounted for. To include the time to reach each state is complex as the exact times of state entry are unknown (interval censoring).

Censoring was used to account for people who were lost to follow-up or alive at the end of the study but in an unknown state. For such individuals, their likelihood was taken as a weighted sum of likelihoods through all possible values for the unobserved state. Forward transitions, which represent movement to a more impaired cognitive state, were allowed between adjacent cognitive states and from all cognitive states to death. A back transition or cognitive recovery was allowed from the mildly impaired state to the non-impaired state. However, due to a lack of observed transitions, it was assumed that recovery from moderate-to-severe impairment was not possible. Where such transitions were observed, they were treated as misclassifications.

To account for measurement error, a hidden Markov Model was specified where state misclassification was allowed to occur at each cognitive interview although independence is assumed between individuals. Initial state probabilities were estimated from the data, taking into account misclassification.

For each multi-state model, parameter robustness was examined by running the models from two different sets of starting values for the transition intensity matrix. The Broyden–Fletcher–Goldfarb–Shanno (BFGS) optimisation method was used to maximise the likelihood. Likelihood ratio tests were use to compare models after the inclusion of each cognitive lifestyle variable; the best fitting model contained all three predictors of cognitive reserve – education, occupation, and social engagement.

For the best fitting model that incorporated all three predictors, life expectancies were calculated. Total residual life expectancy is defined as the sum of occupancy times in each living state. These are calculated by using numerical integration (mid-point rule). Piece-wise hazards (of duration three months) are defined to account for the changing risk of transitions by age. That is, for each three month prediction interval the transition intensity is assumed to be constant. The predicted probabilities of being in each state at the end of the interval were calculated using the pmatrix.msm command from the msm package. The pmatrix.msm command was also used to obtain confidence intervals. These were calculated by simulating 500 random vectors from an asymptotic multivariate normal distribution of the maximum likelihood estimates and then calculating the subsequent transition probability matrices.

**References**

Jackson CH. Multi-State Models for Panel Data: The msm Package for R. (2011) Journal of Statistical Software 38: 1-29.
