# Supplementary material for: Active Cognitive Lifestyle Is Associated with Positive Cognitive Health Transitions and Compression of Morbidity from Age Sixty-Five
Source: PLoS One. 2012 Dec 12;7(12):e50940. doi: 10.1371/journal.pone.0050940 (PMC3521012; doi:10.1371/journal.pone.0050940)
Supplement: Table S1 — Hazard ratios and 95% confidence intervals for the individual effects of education, mid-life occupation and late-life social engagement on cognitive decline and mortality. Table S1 Legend. State 1 (no impairment): MMSE 27–30, State 2 (mild impairment): MMSE 23–26, State 3 (moderate-to-severe impairment): MMSE 0–22. (DOC) [file pone.0050940.s003.doc]

Table S1. Hazard ratios and 95% confidence intervals for the individual effects of education, mid-life occupation and late-life social engagement on cognitive decline and mortality.

|  | Education model | | Occupation model | | Social engagement model | |
| --- | --- | --- | --- | --- | --- | --- |
|  | Education | Education | Occupational complexity | Occupational complexity | Social engagement | Social engagement |
| Transition | (Medium vs. Low) | (High vs. Low) | (Medium vs. Low) | (High vs. Low) | (Medium vs. Low) | (High vs. Low) |
| State 1 - State 2 | **0.6 (0.5, 0.8)** | **0.4 (0.3, 0.6)** | **0.8 (0.6, 1.0)** | **0.6 (0.5, 0.7)** | 0.9 (0.8, 1.2) | 0.9 (0.7, 1.0) |
| State 1 - Death | 1.0 (0.8, 1.1) | 1.0 (0.9, 1.2) | 1.1 (0.9, 1.3) | 1.0 (0.8, 1.2) | 1.0 (0.9, 1.2) | 0.9 (0.7, 1.0) |
| State 2 - State 1 | **2.5 (1.3, 4.6)** | **4.5 (2.2, 8.9)** | 2.2 (0.9, 5.5) | **3.3 (1.4, 8.0)** | 0.8 (0.3, 1.8) | 1.9 (0.9, 3.7) |
| State 2 - State 3 | 0.9 (0.8, 1.1) | 1.1 (0.8, 1.3) | 1.0 (0.8, 1.2) | 1.0 (0.8, 1.2) | 0.9 (0.7, 1.0) | **0.7 (0.6, 0.9)** |
| State 2 - Death | 1.0 (0.7, 1.3) | 0.8 (0.4, 1.4) | 1.0 (0.7, 1.3) | 0.9 (0.6, 1.1) | 0.9 (0.7, 1.2) | 0.8 (0.6, 1.1) |
| State 3 - Death | 1.1 (1.0, 1.2) | **1.3 (1.2, 1.5)** | 1.0 (0.9, 1.1) | **1.2 (1.1, 1.3)** | 1.0 (0.9, 1.1) | 1.0 (0.9, 1.1) |
